# Fatal fulminant cryptococcemia complicating sarcoidosis: Is it to be expected?

**DOI:** 10.1016/j.mmcr.2018.08.006

**Published:** 2018-08-16

**Authors:** Patrizia Posteraro, Katia Vacca, Massimiliano Celi, Giulia De Angelis, Antonietta Vella, Maurizio Sanguinetti, Brunella Posteraro

**Affiliations:** a GVM Ospedale San Carlo di Nancy, Laboratorio di Analisi Cliniche e Microbiologiche, Via Aurelia 275, 00165 Rome, Italy; b GVM Ospedale San Carlo di Nancy, Unità di Medicina, Via Aurelia 275, 00165 Rome, Italy; c Fondazione Policlinico Universitario A. Gemelli IRCCS, Università Cattolica del Sacro Cuore, Istituto di Microbiologia, Largo Gemelli 8, 00168 Rome, Italy; d Fondazione Policlinico Universitario A. Gemelli IRCCS, Università Cattolica del Sacro Cuore, Istituto di Patologia Medica e Semeiotica Medica, Largo Gemelli 8, 00168 Rome, Italy

**Keywords:** *Cryptococcus neoformans*, Cryptococcemia, Sarcoidosis, Capsular antigen, Diagnostic biomarkers

## Abstract

Cryptococcosis may be a life-threatening complication of sarcoidosis. We describe a case of cryptococcemia that rapidly progressed toward fatality without apparent other sites of infection. We discuss on the importance of serum cryptococcal polysaccharide antigen testing for identifying at-risk patients who might benefit from timely diagnosis and treatment of cryptococcosis.

## 1 Introduction

Cryptococcosis, an opportunistic infection caused by the encapsulated yeast *Cryptococcus neoformans*, is a rare but severe complication of sarcoidosis [1]. In a series of 40 sarcoidosis patients with cryptococcal meningitis — the most frequent manifestation of cryptococcosis — identified recently in the literature, 43% (17/40) had a delayed diagnosis, mainly because of initial suspicion of neurosarcoidosis [1].

As currently understood, with the advent of effective antiretroviral therapy, the majority of cryptococcosis cases in nations as ours occurs among non-HIV-infected patients [2], especially in those who have a co-existing immunocompromised state [3]. In these patients, lack of suspicion for, and/or delay in diagnosis of cryptococcosis may increase the likelihood of unfavorable outcome [4]. In a contemporary U.S. population-based study, HIV-negative patients (including transplant recipients) displayed higher mortality rates for cryptococcosis than HIV-positive patients [5].

We describe a case of culture-documented cryptococcemia complicating sarcoidosis, which rapidly progressed toward fatality in the absence of apparent sites of infection except for bloodstream. Concomitant to positive culture, the patient’s serum cryptococcal polysaccharide capsular antigen (CrAg) titer was high (>1:512). We feel that a serologic CrAg screening and timely antifungal therapy at the time of evaluation for evolving sarcoidosis in our patient could have prevented the yeast dissemination in the blood. This case emphasizes the risk for severe or life-threatening cryptococcosis in non-HIV infected, non-transplant (NHNT) patients [5].

## 2 Case

A 75-year-old man with a history of multi-organ sarcoidosis, for which he received corticosteroid therapy (methylprednisolone, 4 mg daily) in the last two years, presented with fatigue, dyspnea, and lower limb edema and pain (day 0). He also suffered of diabetes mellitus and chronic renal failure. Over the last months, he noticed fever for which more hospital admissions were required. During its last hospitalization (on day -45), he experienced a bloodstream infection caused by *Pseudomonas aeruginosa*, which physicians successfully treated with levofloxacin. A chest x-ray revealed pulmonary infiltration with lymphadenopathy, while a chest CT revealed multiple nodules within the lung parenchyma, without pleural effusion, which physicians attributed to an evolving pulmonary sarcoidosis picture. Lung cytology examination did not show abnormal findings. For this reason, physicians increased the methylprednisolone dosage to 16 mg daily.

On examination, the temperature was 36.7 °C (98 °F), while prominent laboratory values included lymphocytopenia of 900 cells/µL, creatinine of 1.73 mg/dL, C-reactive protein of 83 mg/L, and procalcitonin of 2.5 ng/L. The last two values rapidly increased to 160 mg/L and 14 ng/mL, respectively. Bacterial bloodstream infection was suspected and broad-spectrum antibiotic therapy with meropenem and teicoplanin was initiated. Because of his worsening functional status, physicians decided to transfer the patient to the ICU, where he was intubated. On day +3, a tracheal aspirate fluid culture yielded *Candida albicans*, whereas a blood culture was positive for yeasts at the microscopic Gram-stain observation. Based on these findings, the patient immediately initiated antifungal therapy with fluconazole (400 mg daily).

On day +5, the yeast isolated from the patient’s blood was identified as *C. neoformans*, and the serum positive titers for CrAg (≥1:4096) confirmed disseminated cryptococcal disease. Physicians did not perform lumbar puncture to rule out asymptomatic CNS involvement, because they judged it as contraindicated by the worsened patient’s conditions. Anyway, physicians changed fluconazole to liposomal amphotericin B (80 mg daily) on day +6. Despite appropriate institution of antifungal therapy, the patient died for septic shock on day +10.

We initially identified the patient’s blood isolate as *C. neoformans* by the automated VITEK^®^ 2 system (bioMérieux, Marcy-l'Étoile, France) using the YST ID card, which contains biochemical/enzymatic substrates for rapid and accurate identification of a broad range of pathogenic yeasts, including 5 *Cryptococcus* species (*C. albidus*, *C. laurentii*, *C. neoformans*, *C. terreus*, and *C. uniguttulatus*). This result was communicated to the physician for a prompt use of it. Subsequently, we confirmed the isolate’s identification by the MALDI (matrix-assisted laser desorption ionization) BioTyper^®^ system (Bruker Daltonics, Bremen, Germany) according to the protocol previously developed by us [6]. Briefly, a 1-µL aliquot of the protein extract sample obtained from the isolate was spotted onto a MALDI target plate and was subjected to measurements with a microflex LT mass spectrometer (Bruker Daltonics). Spectra from sample’s triplicates were generated and used for pattern matching against the UCSC yeast database (an in-house “fast” yeast library purposely created with a fast sample preparation procedure) [7], using the BioTyper software package (version 3.1.66). According to criteria proposed by the manufacturer’s (log(score) values, ≥ 2.0 for identification at the species level; ≥ 1.7 and ≤ 2.0, for identification at least at the genus level, and values < 1.7, for identification not reliable), we successfully identified the patient’s isolate as *C. neoformans* based on a log(score) of 2.427. Additionally, hierarchical cluster analysis was conducted with the integrated statistical tool Matlab 7.1 of the BioTyper software package, to generate a similarity dendrogram based on graphical distance values between the present study’s isolate and other studies’ isolates [6]. This analysis showed that the patient’s isolate separated with other isolates within the *C. neoformans* var. *grubii* (serotype A) VNI (genotype) cluster. Antifungal susceptibility testing of the patient’s isolate was performed as previously described [8], using the Sensititre YeastOne (Thermo Fisher Scientific, MA) plate. The concentrations of the antifungals ranged from 0.12 to 8 μg/mL for amphotericin B, 0.06–64 μg/mL for flucytosine, 0.015–8 μg/mL for anidulafungin, 0.008–8 μg/mL for caspofungin, micafungin, voriconazole, and posaconazole, 0.12–256 μg/mL for fluconazole, and 0.015–16 μg/mL for itraconazole. Apart from echinocandins — anidulafungin, caspofungin, and micafungin — to which *C. neoformans* is considered intrinsically resistant, all antifungals showed low minimum inhibitory concentration values. Using the epidemiological cut-off values established for *C. neoformans* VNI and fluconazole (8 μg/mL), itraconazole (0.5 μg/mL), posaconazole (0.25 μg/mL), and voriconazole (0.25 μg/mL) [9], the patient’s isolate was defined as wild-type for the susceptibility to azole antifungal agents.

## 3 Discussion

Cryptococcosis, typically a disease of immunocompromised hosts, has a wide array of clinical presentations that makes its diagnosis particularly challenging [10]. In hosts with impaired cell-mediated immunity (i.e., not necessarily HIV-infected hosts), cryptococcal pneumonia is usually symptomatic and, sometimes, progresses rapidly to acute respiratory distress syndrome [10]. This accompanies with radiologic findings, including well-defined single or multiple noncalcified nodules and pulmonary infiltrates [2], which may mimic those frequently seen in pulmonary sarcoidosis [11]. Dyspnea was the sole symptom attributable to pulmonary cryptococcosis in our patient. Among 302 patients with cryptococcosis studied by Pappas et al. [2], transplant recipients and NHNT patients were less likely to have CNS involvement and cryptococcemia compared to HIV-positive patients. However, subanalysis of that cohort showed that bloodstream involvement and the mean time to diagnosis differed significantly between immunocompromised and non-immunocompromised patients (*P* < 0.001 for both comparisons) [2].

Poor pro-inflammatory immune responses to *C. neoformans*, such as those elicited in patients with advanced HIV infection, may lead to life-threatening disease [12]. In patients with sarcoidosis, alveolar macrophages are hyper-reactive, and the T-cell immune response is exaggerated because of the concomitant influx of CD4-T cells and absence of regulatory T-cell mechanisms in sarcoid granulomas [11]. Consequently, lymphocyte counts in peripheral blood of sarcoidosis patients are usually low, and lymphocytopenia correlates with disease severity.

This case shows that disseminated cryptococcosis, even without apparent CNS involvement, is a serious threat for patients with multiple risk factors, including corticosteroid therapy, sarcoidosis, lymphocytopenia, and renal failure [10]. With the benefit of hindsight, careful evaluation of the risk for cryptococcosis in our patient should have driven the treating clinician to go beyond the suspicion of bacterial infection. However, invasive fungal infections are often neglected, while awareness of their frequency and clinical relevance is needed to help the management of these highly deadly mycoses [13]. Thus, lack of both risk factor assessment and specific disease symptoms may lead the clinician to fail in making an early diagnosis of cryptococcemia or cryptococcal meningitis. This is despite the availability of rapid diagnostic tools, such as detection of CrAg in serum and cerebrospinal fluid [14].

It was appreciated that CrAg testing by latex agglutination or ELISA has an overall sensitivity and specificity of 93–100% and 93–98%, respectively, and it can be positive before the growth of viable cryptococcal colonies in culture [14]. The detection of CrAg was greatly improved with the advent of point-of-care diagnostic assays, such as a lateral flow assay for use in serum and other sample types — including whole blood from finger stick samples [10]. However, a “screen and treat” approach based on testing for cryptococcal antigenemia followed by preemptive administration of antifungals is not part of standard practice in non-HIV infected patients [3], while it is a consolidated practice in resource-limiting settings with high incidence of HIV and cryptococcal diseases [14].

In conclusion, our case underlines the importance of recognizing sarcoidosis patients who are at high risk for cryptococcal disease to avoid the harmful consequences related to delayed or missed diagnosis. As a high CrAg titer is associated with a large burden of yeasts that might be critical to the disease outcome, timely using this biomarker allows to identify which patients might benefit from diagnosis and treatment of cryptococcosis.
